# A Hand-Book of Therapeutics (Ringer)

**Published:** 1889-05

**Authors:** 


**A Handbook of Therapeutics.** By SIDNEY RINGER, M. D., Professor of the Principles and Practice of Medicine in University College, Physician to University Hospital (London). Twelfth edition, 8vo, pp. xi., 524. New York: William Wood & Company, 56 and 58 Lafayette place. 1889.

When a work has reached its twelfth edition, it is presumed that its value is so well established, and the profession has become so familiar with it, that very little need be said by the reviewer either in praise or blame. The book before us merits only the former. Sidney Ringer is a household name in professional circles on this side of the Atlantic, and his "Therapeutics" has become classic.

The present edition, as might be expected, is the most complete that the author has issued, and contains information on most of the newer drugs, as well as much fresh knowledge on the older. He follows the same general plan as in former editions, dwelling particularly on the indications for the use of drugs in disease. This makes the work of great value to the general practitioner, and may be considered as one of its strongest points. Here may be obtained just the information sought upon any particular agent, without circumlocution or verbosity.

The author emphasizes the fact in his introduction, that too little attention has of late been paid to the detection and appreciation of symptoms, a defect in the training of students that he seeks to correct. Not denying the importance of physical signs, and a knowledge of their interpretation, he still insists that, as we, in the majority of cases, are compelled to treat the secondary effects of a malady rather than the primary disease, it is of the first importance that the symptoms shall receive weighty consideration. The former are chiefly useful in diagnosis, whereas the latter guide to proper prognosis and treatment. This, it seems to us, is the only rational ground on which the therapeutist can lay a strong foundation for the proper application or administration of his remedies, and, therefore, the most direct avenue through which to expect reasonably prompt results therefrom.

The first thirty-five pages of the volume are devoted to the consideration of the objective symptoms of disease, as indicated by the tongue, the pulse, the skin, the temperature of health and disease, fever, and dropsy; the next fifty pages treat of therapeutic agents other than drugs, *e.g.*, cold, heat, baths, fomentations, counter-irritation, etc., etc.; finally, in the bulk of the volume, 381 pages, the materia medica proper receives a most masterly exposition, each drug being allotted space commensurate with its importance, and the knowledge that has yet been obtained concerning its therapeutical value. It is replete with the latest knowledge concerning the recent additions to the already long list of therapeutic agents.

The ever-changing nature of materia medica renders it necessary that frequent editions of therapeutical works like this shall be put forth, and makes it equally requisite that those who would keep pace with progress in this direction, shall supply themselves with such new editions from time to time. This is equally imperative upon the teacher and practitioner.

A dietary for invalids extends through eleven pages of the book, that cannot fail to prove useful to physician and nurse, and a comprehensive index of therapeutic agents and diseases contribute to make it altogether the best edition we have yet seen of this standard work. The publishers deserve a meed of praise for their part of the production.

